# Semantic Radicals Contribute More Than Phonetic Radicals to the Recognition of Chinese Phonograms: Behavioral and ERP Evidence in a Factorial Study

**DOI:** 10.3389/fpsyg.2017.02230

**Published:** 2017-12-19

**Authors:** Xieshun Wang, Meng Pei, Yan Wu, Yanjie Su

**Affiliations:** ^1^School of Psychological and Cognitive Sciences, Beijing Key Laboratory of Behavior and Mental Health, Peking University, Beijing, China; ^2^School of Psychology, Northeast Normal University, Changchun, China

**Keywords:** semantic radical, phonetic radical, functional validity, Chinese phonogram, ERP

## Abstract

The Chinese phonograms consist of a semantic radical and a phonetic radical. The two types of radicals have different functional contributions to their host phonogram. The semantic radical typically signifies the meaning of the phonogram, while the phonetic radical usually contains a phonological clue to the phonogram’s pronunciation. However, it is still unclear how they interplay with each other when we attempt to recognize a phonogram because previous studies rarely manipulated the functionality of the two types of radicals in a single design. Using a full factorial design, the present study aimed to probe this issue by directly manipulating the functional validity of the two types of radicals in a lexical decision task with both behavioral and event-related potential (ERP) measurements. The results showed that recognition of phonograms which were related to their semantic radicals in meaning took a shorter reaction time, showed a lower error rate, and elicited a smaller P200 and a larger N400 than did recognition of those which had no semantic relation with their semantic radicals. However, the validity of phonetic radicals did not show any main effect or interaction with that of semantic radicals on either behavioral or ERP measurements. These results indicated that semantic radicals played a dominant role in the recognition of phonograms. Transparent semantic radicals, which provide valid semantic cues to phonograms, can facilitate the recognition of phonograms.

## Introduction

The character is the primary perceptual and writing unit of Chinese (Feldman and Siok, 1999; Zhang et al., 2009). A large number of Chinese characters are phonograms, which are composed of a semantic radical (usually on the left) and a phonetic radical (usually on the right) (Lee et al., 2006; Wang, 2006). The two types of radicals have different functional contributions to the phonograms. The semantic radical usually implies the meaning of its host phonogram, but the phonetic radical often offers a phonological clue for the pronunciation of its host phonogram (e.g., the phonogram, *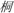, tong2: tung tree*, whose semantic radical is *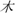, mu4: wood*, and phonetic radical is *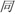, tong2: together/same*). The configuration of phonogram means both the semantic and phonological information is embedded at the sub-lexical level of a phonogram (Williams and Bever, 2010).

Converging evidence has showed that the cognitive process of compound characters (e.g., phonograms) involved the activation of their own radicals, and was modulated by features of the radicals (Feldman and Siok, 1997, 1999; Taft and Zhu, 1997; Taft et al., 1999; Ding et al., 2004; Lee et al., 2006; Wu et al., 2012). Previous research demonstrated that both phonological and semantic information in phonetic radicals could be activated during the recognition of phonograms. For example, using primed naming task, Zhou and Marslen-Wilson (1999a) found that a phonogram (e.g., *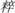, cui4: pure*, whose phonetic radical is *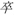, zu2: soldier*) could facilitate the recognition of its phonetic radical’s homophone (e.g., *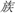, zu2: race*). Another primed naming study by Zhou and Marslen-Wilson (1999b) also revealed a facilitative priming effect that a phonogram (e.g., *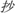, chao1: transcribe*, whose phonetic radical is *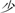, shao3: few*) could promote the recognition of characters semantically related to its phonetic radical, even an antonym (e.g., *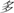, duo1: many*). This effect was further verified by an event-related potential (ERP) study (Lee et al., 2006). Interestingly, Zhou et al. (2013) found that both semantic information and phonological information in semantic radicals could be activated during phonogram recognition using the same primed naming paradigm as Zhou and Marslen-Wilson (1999a,b). For example, the phonogram, *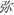, mi2: full*, with the semantic radical *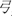, gong1: bow*, could facilitate the recognition of *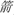, jian4: arrow*, which is semantically related to *
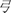
*; and the phonogram, *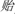, yi2: present*, with the semantic radical *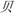, bei4: shell*, could facilitate the recognition of *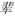, bei4: generation*, which is a homophone of *
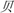
*.

Recently, since the semantic and phonetic radicals have different functions, some researchers turned to compare the processing of semantic and phonetic radicals to examine whether their distinctive functions would elicit different processing patterns. Unfortunately, the results of these studies are mixed, with some studies reporting that the effect of phonetic radicals was stronger than that of semantic radicals (Wang, 2006; Hung et al., 2014), and still others assuming that the semantic radicals played a predominant role (Williams and Bever, 2010). For example, using an apparent motion (AM) detection task, Wang (2006) asked participants to judge which of the two radicals of phonograms was displaced toward the side, top, or bottom. Moreover, additional recognition demands (naming in Experiment 2; lexical decision in Experiment 3) were also imposed. The results showed that participants detected the AM of phonetic radicals more efficiently than semantic radicals when the token frequencies of their host phonograms were low. These findings were interpreted as a bias toward the orthographic features of phonetic radicals during the recognition of phonograms. In a magnetoencephalography (MEG) study, Hung et al. (2014) traced the dynamics of priming effect induced by the repetition of phonetic radicals in a homophone judgment task, and the time course of priming effect caused by the repetition of semantic radicals in a synonym judgment task. The results only showed that the repetition effect of the phonetic radical was significant in the M170, M250, and M350 time windows. The authors claimed that the phonetic radicals might play a predominant role in recognition of phonograms on the basis of these MEG results. However, their behavioral results showed that the repetition of phonetic and semantic radicals could both have impacts on the recognition of phonograms. In contrast, Williams and Bever (2010) compared the effect of semantic and phonetic radical in a single design (Experiment 3). Specifically, they carried out a modified lexical decision task in which either the semantic radical or the phonetic radical in a visual phonogram was blurred. The phonograms with a blurred semantic radical were found to be harder to recognize than were those with a blurred phonetic radical, which suggested that the semantic radicals, not the phonetic radicals, were more pivotal in the recognition of phonograms. However, these studies did not provide solid evidence for the functional interaction between the semantic and phonetic radicals because most of them manipulated their functionality in separate experiments, and Williams and Bever (2010) had a methodological drawback. Blurring character may impede the normal perceptual processing in the initial lexical access because the participants have to focus more on identifying the blurred radicals. In particular, semantic radicals are usually smaller in size than phonetic radicals (Wang, 2006), hence blurred semantic radicals are visually harder to recognize.

Therefore, in the present study, we adopted a full factorial design and manipulated the functional validity of both semantic radicals (Transparency) and phonetic radicals (Regularity) in a lexical decision task. A semantic radical (S) that is semantically related to its host phonogram is considered transparent (S+), or else is considered opaque (S-). Similarly, a phonetic radical (P) is regarded regular (P+) if it has the same pronunciation as its host phonogram, otherwise it is regarded irregular (P-). By Transparency and Regularity, all phonograms are divided into four types. The first type of phonograms have a transparent semantic radical and a regular phonetic radical (S+P+ phonograms). The second type have a transparent semantic radical but an irregular phonetic radical (S+P- phonograms). The third type have an opaque semantic radical but a regular phonetic radical (S-P+ phonograms). Finally, the fourth type have an opaque semantic radical and an irregular phonetic radical (S-P- phonograms). For the lexical decision task in the present study, we used pseudo-characters as fillers and asked participants to judge whether the stimulus was a true character or not. The lexical decision task is advantageous in that it is a strategy-neutral reading task (Williams and Bever, 2010), allowing us to compare the processing of the semantic and phonetic radicals on a fair basis. In contrast, the semantic judgment task and the homophone judgment task would be likely to enhance the sub-lexical retrieval of semantic or phonological information, respectively (Zhang et al., 2009). However, our lexical decision task was very different from the one used in Williams and Bever (2010), as we did not blur the radicals so that participants could read the characters in a relatively naturalistic way.

The present study compared the different processing patterns in recognizing the four types of phonograms using both behavioral and ERP measures. The measurement of reaction time reflects the accumulated outcome of all processing steps before the behavioral response to a language task, while the ERP measurement with a millisecond temporal resolution is well-suited to uncovering the time course of cognitive processing from the onset of linguistic stimulus (Lee et al., 2007). In order to minimize any potential influence from decision-related responses on electroencephalography (EEG) signals (Lee et al., 2006), we recorded behavioral (Behavioral Experiment) and ERP (ERP Experiment) data in two separate experiments. We asked the participants in Behavioral Experiment to make an immediate response, but the ones in ERP experiment to make a delayed response so that they pressed the button 1100 ms after stimulus onset, thus the reaction times were not so meaningful in ERP Experiment.

There has been hardly any study employing a full factorial design to explore the interaction between semantic and phonetic radicals. Nonetheless, we hypothesized that if the semantic and the phonetic radicals did not contribute equally to the processing of the host phonogram at the functional level, then in a task under time pressure such as lexical decision, the secondary contributor would influence the processing only when the primary contributor did not contain valid information (i.e., when the phonetic radical was irregular or the semantic radical was opaque). As for electrophysiological indicators, we focused on two ERP components, P200 and N400, as the studies on the recognition of single Chinese characters suggested. The P200, a positive wave peaking around 200 ms post-stimuli onset, was believed to reflect the early extraction of information from the radicals; and the N400, characterized by a negative peak at around 400 ms post-stimuli onset, was a major index of post-lexical processing (Hsu et al., 2009; Wu et al., 2012; Yum et al., 2014). These studies had demonstrated that a radical which elicited a smaller P200 often evoked a larger N400 afterward. A smaller P200 indicated easier extraction of information in radicals, and easier extraction would lead to a greater activation of the radicals’ representations in the post-lexical phase, and therefore led to a strong stimulation on N400. We attempted to examine how the semantic and phonetic radicals interplay with each other with four kinds of phonograms embedding sub-lexical functional information of varying validity.

## Behavioral Experiment

### Methods

#### Participants

Thirty right-handed undergraduates or post-graduates gave informed consent to participate in the experiment (mean age = 21 years, *SD* = 2.8, range = 17∼27, 21 females). All of them were native speakers of Mandarin and had normal or corrected-to-normal vision.

#### Materials

All of the phonograms used in the present study have their semantic radical on the left and their phonetic radical on the right, and are called SP phonograms. SP phonograms are the most common phonograms (making up around 70%) (Hsiao and Shillcock, 2006). Also, the majority of phonograms used in previous studies were SP phonograms. In the present study, four sets of SP phonograms (range of token frequency: 1.81∼359.07 per million; *M* = 72.47, *SD* = 87.94) were selected from an online modern Chinese database (Chinese Text Computing at http://lingua.mtsu.edu/chinese-computing/, by Dr. Jun Da) (Li et al., 2011; Zhao et al., 2013). The four sets were used respectively for the four experimental conditions: S+P+, S+P-, S-P+, and S-P- (see **Table 1**). S+P+ and S+P- conditions each had 37 items, and S-P+ and S-P- conditions each had 32 items. Twenty undergraduates (14 females) who were not to participate in the lexical decision experiment completed a pilot test for the rating of Transparency and Regularity. All the participants were native Mandarin speakers. First, the participants were asked to rate the semantic correlation between the semantic radicals and their host phonograms (i.e., degree of transparency) on a 1-to-5 scale (1 = extremely unrelated, 5 = extremely related). Descriptive statistics about degree of transparency of the four conditions are as follows: S+P+: *M* = 3.68, *SD* = 0.22, range = 3.25∼4.20; S+P-: *M* = 3.64, *SD* = 0.30, range = 3.25∼4.35; S-P+: *M* = 2.29, *SD* = 0.28, range = 1.70∼2.75; S-P-: *M* = 2.19, *SD* = 0.31, range = 1.70∼2.85. A Transparency (Transparent vs. Opaque) × Regularity (Regular vs. Irregular) analysis of variance (ANOVA) performed on the mean degree of transparency scores only revealed an effect of Transparency [*F*(1,134) = 883.807, *p* < 0.001, $\eta_{p}^{2}$ = 0.868], but no effect of Regularity [*F*(1,134) = 1.619, *p* = 0.131, $\eta_{p}^{2}$ = 0.017] or interaction between the two variables [*F*(1,134) = 0.160, *p* = 0.542, $\eta_{p}^{2}$ = 0.003]. Second, these participants were asked to judge whether the phonetic radicals were pronounced identically with their host phonograms. The results showed that all of the participants reported the phonetic radicals in both S+P+ and S-P+ conditions were pronounced identically to their host phonograms, whereas the phonetic radicals in S+P- and S-P- conditions were all reported to be pronounced differently. Other relevant features (i.e., token frequency of phonogram; stroke number of phonogram; stroke number of semantic radical; stroke number of phonetic radical) of the four sets are showed in **Table 1**. Four Transparency (Transparent vs. Opaque) × Regularity (Regular vs. Irregular) ANOVAs on the four extraneous variables did not find any significant difference, *p*s > 0.1. Finally, we designed 138 left–right structured pseudo-characters as fillers for the lexical decision task. Each pseudo-character was created by combining two radicals from existing characters in their legal positions (left or right side). Therefore, the pseudo-characters were orthographically regular. Readers would not know just from seeing one part of the character that it was an illegal character. Nevertheless, all pseudo-characters were unpronounceable and meaningless. For example, the pseudo-character, *
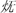
*, was created with the left radical *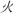, huo3: fire*, and the right radical *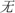, wu2: nothing*.

**Table 1:** Examples and characteristics of stimuli for each experimental condition.

Condition	S+P+	S+P-	S-P+	S-P-
Character example	* 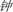 (bell)*	* 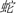 (snake)*	* 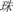 (bead)*	* 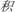 (to amass)*
Set size	37	37	32	32
Token frequency of phonogram	70.62 (83.59)	73.71 (82.51)	71.81 (94.57)	73.85 (95.94)
Stroke number of phonogram	9.73 (2.12)	9.84 (2.09)	9.38 (2.38)	9.09 (1.84)
Stroke number of semantic radical	3.68 (1.00)	3.65 (1.06)	3.22 (1.01)	3.53 (1.11)
Stroke number of phonetic radical	6.05 (1.79)	6.19 (1.84)	6.16 (1.94)	5.56 (1.65)

*Standard deviations in parentheses.*

#### Procedure

Participants were seated in a comfortable chair approximately 60 cm from a CRT computer screen with a black background. All of the stimuli were in white. Each trial began with a fixation cross for 500 ms at the center of the screen, followed by a blank screen for a jittered duration between 500∼700 ms. Next, either a phonogram or a pseudo-character (visual angle: 1.9° × 1.9°) was presented. The participants were asked to press the button immediately when they saw the stimulus to judge whether it was a true character or not. The stimuli would disappear either upon the participant’s response or 2500 ms after onset if no response was detected. The phonograms across the four conditions and the pseudo-characters were presented in random order. The key configuration was counterbalanced across participants so that half of them pressed the right “Alt” key to give a “Yes” response and pressed the left “Alt” key to give a “No” response, while the other half followed the reverse configuration. Finally, a blank screen for 600 ms was presented serving as the interval before the next trial. Before the formal experiment, participants performed 12 practice trials to ensure that they fully understood the procedure.

### Results

The means of RTs (ms) and accuracy (ACC) across four conditions were showed in **Table 2**. However, for the main analysis, RTs and ACC were instead analyzed using a linear mixed-effect model (LMM), with the R Package *lme4* (Version 1.1-12; Bates et al., 2015). The package was retrieved from http://cran.r-project.org/package=lme4, and the current version is 1.1-13.

**Table 2:** RTs (ms) and ACC (%) across four conditions in Behavioral Experiment.

Condition	S+P+	S+P-	S-P+	S-P-
RTs	552 (59)	558 (65)	575 (65)	570 (72)
ACC	97.2 (16.5)	98.1 (13.6)	96.4 (18.5)	96.3 (18.8)

*Standard deviations in parentheses.*

#### RTs

Trials with an incorrect response, with no response given within 2500 ms, or with a reaction time exceeding ± 3 *SD*s from the grand mean were all discarded from analysis (4.9%). The two manipulated variables and their interaction (Transparency × Regularity) were entered as fixed factors, the subject-specific and item-specific intercepts as crossed random factors. Additionally, the model included the subject-specific random slopes for each of the two fixed factors and their interaction. Since distribution of the RTs was positively skewed, we entered the log-transformed RTs into the model. Estimated effect sizes (*b*), standard errors (*SE*), *t*-values, and *p*-values were reported.

Because the interaction between Transparency and Regularity was not significant (*b* = -0.018, *SE* = 0.021, *t* = -0.850, *p* = 0.39), two difference contrasts were applied to determine the estimated sizes of Transparency effect (Opaque minus Transparent) and Regularity effect (Irregular minus Regular). The results showed that recognition in the Opaque condition was slower than that in the Transparent condition (*b* = 0.032, *SE* = 0.011, *t* = 3.00, *p* = 0.003), but the effect of Regularity was not significant (*b* = 0.012, *SE* = 0.011, *t* = 0.113, *p* = 0.91).

#### ACC

We coded accuracy into a categorical variable (correct = 1, incorrect = 0), and ran a logistic LMM for it. The fixed factors and crossed random factors in the model for RTs were retained. However, the subject-specific random slopes for each of the two fixed factors and their interaction were removed since the current model failed to converge. The values of *b* and *SE* in logits, *z*-values, and *p*-values were reported.

The results showed that the interaction between the two fixed factors was not significant (*b* = -0.385, *SE* = 0.468, *z* = -0.823, *p* = 0.41). Similarly, two difference contrasts were used to determine the estimated sizes of Transparency effect and Regularity effect. The results showed only a marginally significant effect of Transparency (*b* = -0.459, *SE* = 0.234, *z* = -1.96, *p* = 0.05). The response in the Opaque condition was less accurate than that in the Transparent condition.

Overall, the behavioral results indicated that semantic radicals were more prominent in phonogram recognition than phonetic radicals, and the recognition of phonograms with a transparent semantic radical was faster and more accurate than that of phonograms with an opaque semantic radical.

## ERP Experiment

### Methods

#### Participants

Twenty right-handed native Mandarin-speaking undergraduates or post-graduates (mean age = 22 years, *SD* = 1.9, range = 18∼25, 17 females) were recruited. All participants had normal or corrected-to-normal vision with no reading difficulties, learning difficulties or history of neurological or psychiatric disorders. All of them gave informed consent before the experiment. Data from two participants were excluded from the final analysis due to excessive artifacts.

#### Materials

Materials used in Behavioral Experiment were also used in ERP Experiment. However, to run enough trials to obtain more reliable ERP data, all materials including the pseudo-characters were presented twice in two separate sessions, one time in each (similar to Wu et al., 2012, 2017).

#### Procedure

**FIGURE 1:**
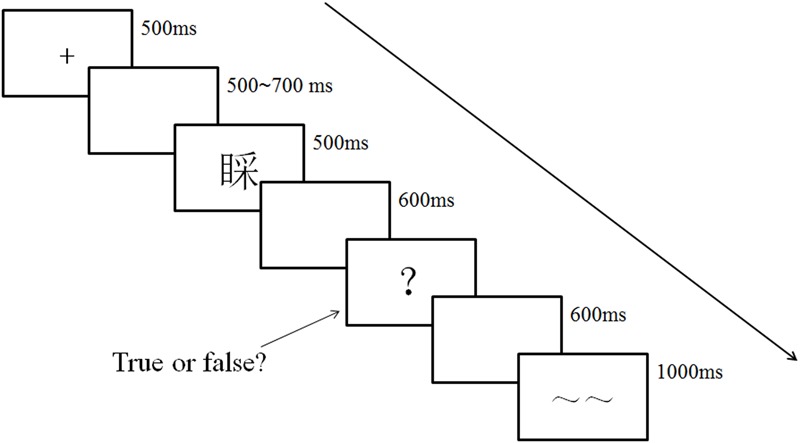
Experimental procedure in ERP Experiment.

In order to avoid interference from motion-related or decision-related EEG activity, we adopted a delayed response procedure, so the procedure in ERP Experiment was a bit different from the one in Behavioral Experiment. As showed in **Figure 1**, the difference was that the stimuli only appeared for 500 ms, followed by a blank screen for 600 ms. Subsequently, a response signal “?” was displayed. The participants were instructed to respond after they saw the response signal. The response signal would disappear upon the participant’s response or 2500 ms after onset with no response detected. Afterward, another blank screen for 600 ms was presented, followed by a blink signal “∼∼” for 1000 ms. Participants were instructed not to blink or move their eyes except when they saw this signal to avoid ocular artifacts in ERP data. All participants were tested individually in a room with electromagnetic shielding and were seated 60 cm from the computer screen. Finally, key configuration was also counterbalanced across participants, and they performed 12 practice trials prior to the formal experiment.

#### ERP Recording and Analyses

The EEG was recorded using a 64-channel Ag/AgCl electrode cap (QuickCap, Neuromedical Supplies, Sterling, VA, United States). The on-line reference was the left mastoid and replaced with the average of the left and the right mastoids as off-line re-reference. The vertical EOG (electrooculogram) was obtained by two electrodes placed above and below the left eye, and the horizontal EOG by two electrodes placed on the outer canthi. The impedance of each electrode was kept below 5 kΩ. The signal was amplified by a SynAmps2^®^ amplifier (Neuroscan, Inc., EL Paso, TX, United States) with a band-pass of 0.05∼100 Hz and continuously digitized at 1000 Hz. The off-line analysis was conducted with the Scan 4.5 software (Neuroscan, Charlotte, NC, United States). The EEG was filtered with a low-pass filter of 30 Hz and segmented into epochs of 700 ms, including a 150 ms pre-stimulus baseline. Ocular artifacts from eye blinks or horizontal movements were automatically corrected using the Scan software. Epochs with amplitudes exceeding ± 80 μV as well as incorrect responses were excluded. The overall rejection rate was 2.82%, and the remaining epochs for each condition were averaged to produce the grand mean waveforms.

Based on visual inspection and the prior ERP studies (Su et al., 2012; Wu et al., 2012; Yum et al., 2014), we identified the ERP-components P200 and N400 both at electrode sites F1, F3, F5, FC1, FC2, FC3, F2, F4, F6, FC2, FC4, and FC6. The time window of P200 was set between 160∼230 ms, and the time window of N400 was 320∼420 ms. For each ERP-component, we pooled the activities over the left hemispheric electrodes F1, F3, F5, FC1, FC2 and FC3, and right hemispheric electrodes F2, F4, F6, FC2, FC4, and FC6. Separate LMMs were used to analyze the pooled data on each hemisphere. In the models, Transparency, Regularity, and their interactions were entered as fixed factors. Since the original data of each participant were averaged over items for each condition, the item-specific intercept was not included as a crossed random factor. Alternatively, we entered the subject-specific intercept and session-specific intercept as crossed random factors. Entering session-specific intercept was to consider the difference in recognition of the items between two sessions. When the interaction between Transparency and Regularity was not significant, the difference contrasts were applied to determine the estimated sizes of Transparency effect (Opaque minus Transparent) and Regularity effect (Irregular minus Regular).

### Results

#### Overview

**FIGURE 2:**
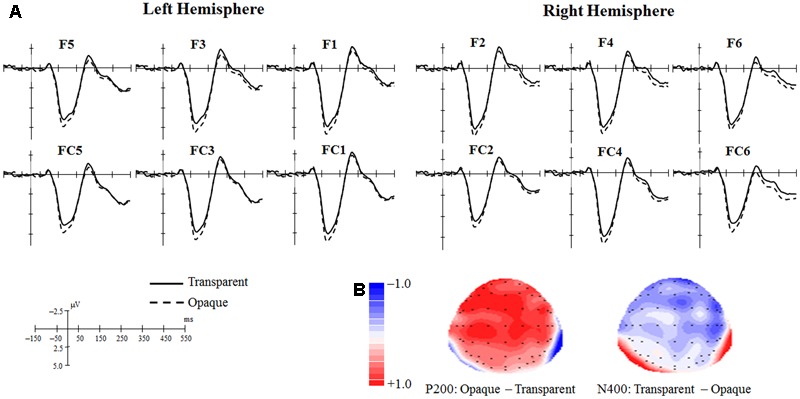
**(A)** Grand mean ERPs of the Transparent condition and Opaque condition in each electrode. **(B)** Topographical maps of scalp voltage showing the differences between the two conditions.

The grand mean ERPs of the Transparent and Opaque conditions in each electrode of interest are plotted in **Figure 2A**. Topographical maps of scalp voltage showing the differences between the two conditions in P200 and N400 time windows are presented in **Figure 2B**. These visualized results implied that the Transparent condition might evoke a smaller P200 and a larger N400 in anterior region than the Opaque condition did.

**FIGURE 3:**
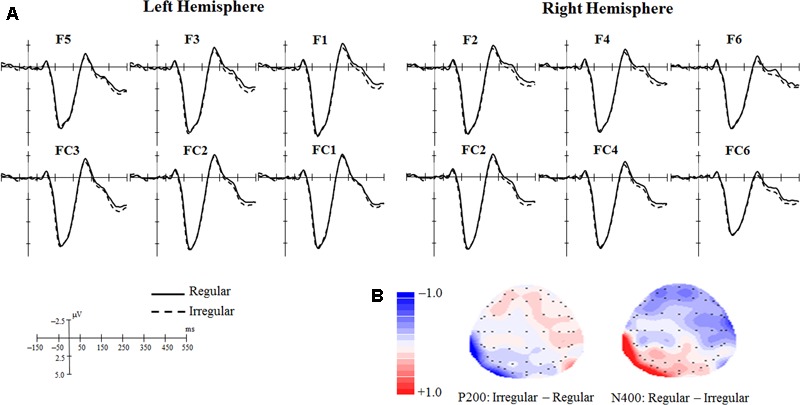
**(A)** Grand mean ERPs of the Regular condition and Irregular condition in each electrode. **(B)** Topographical maps of scalp voltage showing the differences between the two conditions.

The grand mean ERPs of the Regular and Irregular conditions in each electrode of interest are plotted in **Figure 3A**. Topographical maps of scalp voltage showing the differences between the two conditions in P200 and N400 time windows are presented in **Figure 3B**. These visualized results implied that the Regular condition might evoke a larger N400 in anterior region than the Irregular condition did. The results of LMMs are as follows.

#### P200

In the left hemisphere, the interaction between Transparency and Regularity was not significant (*b* = -0.406, *SE* = 0.515, *t* = -0.789, *p* = 0.43). A further analysis using difference contrasts showed only an effect of Transparency (*b* = 0.856, *SE* = 0.257, *t* = 3.33, *p* = 0.001). The Transparent condition yielded a less positive component than the Opaque condition did. However, the effect of Regularity was far from significant (*b* = -0.055, *SE* = 0.257, *t* = -0.213, *p* = 0.831).

The right hemisphere showed an identical pattern of results. The interaction between Transparency and Regularity was not significant, too (*b* = -0.534, *SE* = 0.483, *t* = -1.104, *p* = 0.27). Similarly, only the effect of Transparency was significant (*b* = 0.761, *SE* = 0.242, *t* = 3.15, *p* = 0.002), but not the effect of Regularity (*b* = 0.082, *SE* = 0.242, *t* = 0.339, *p* = 0.735). The Transparent condition elicited a less positive component than the Opaque condition did.

#### N400

In the left hemisphere, neither the interaction nor the main effects of Transparency and Regularity were significant (*p*s > 0.2). In the right hemisphere, the interaction was not significant (*b* = 0.20, *SE* = 0.568, *t* = -0.352, *p* = 0.726). However, a follow-up analysis with difference contrasts showed a significant effect of Transparency (*b* = 0.565, *SE* = 0.283, *t* = 1.997, *p* = 0.048). The component elicited in the Transparent condition was more negative than that in the Opaque condition. Still, the effect of Regularity was insignificant (*b* = 0.167, *SE* = 0.283, *t* = 1.591, *p* = 0.556).

## Discussion

The Chinese phonograms consist of a semantic radical and a phonetic radical, which differ in their functional contributions to their host phonogram. The present study was aimed at examining how the two types of radicals interplayed with each other in the recognition of phonograms. To our knowledge, this was the first factorial study to probe this question by directly manipulating the functional values of both types of radicals. Plus, by manipulating transparency and regularity at the same time and matching items on variables such as the frequency of phonogram that might be relevant to processing, we attempted to further reduce confounding with variables of interest. In a Behavioral Experiment, we found that phonograms with a transparent semantic radical were recognized faster and more accurately than those with an opaque semantic radical. Furthermore, in ERP Experiment, we also found significant effects on both P200 and N400 caused by Transparency of semantic radicals. Phonograms with a transparent semantic radical evoked a smaller P200 and a larger N400 than those with an opaque semantic radical did. As for Regularity of phonetic radicals, we did not find any effect of it on ERP components of interest, reaction time, or error rates.

Theoretically, transparent semantic radicals and regular phonetic radicals are more easily activated than their counterparts, because they can respectively provide valid semantic and phonological cues to their host phonograms. Previous studies which manipulated the two types of radicals in separate experiments had demonstrated that transparent semantic radicals and regular phonetic radicals could both facilitate the recognition of their host phonograms (Chen and Weekes, 2004, Experiment 1; Li and Chen, 1999; Williams and Bever, 2010, Experiments 1, 2; Yum et al., 2014). However, one study which compared the effects of the two types of radicals in a single design showed that the semantic radicals might play a more important role in recognition of phonograms (Williams and Bever, 2010). This indicated that the recognition of phonograms was likely to rely more upon the semantic path in a strategy-neutral task. Using a full factorial design, the present study supported the findings. Although the functional validity of semantic and phonetic radicals were factorially manipulated, only semantic radicals were found to have effects on behavioral and ERP measures. The behavioral results indicated that the transparent semantic radicals could facilitate the recognition of phonograms. The ERP results implied that this facilitation might result from easier activation of transparent semantic radicals. Specifically, the smaller P200 indicated that the information in transparent semantic radicals was easier to be extracted in the early stage of phonogram recognition, and the larger N400 suggested that transparent semantic radicals then triggered a greater activation of representations in the later stage of phonogram recognition. However, the Transparency effect on N400 was observed only in the right hemisphere. This is consistent with the argument of Hsiao and Liu (2010) that the semantic information in SP phonogram would be processed primarily in the right hemisphere.

Yet for all the findings, we should not say that phonological regularity does not have any effect on processing of Chinese characters during reading. There is a consensus that the compound characters and their radicals were represented at multiple levels of one’s mental lexicon: the compound characters were in the lexical level, and radicals were in the sub-lexical level (Taft, 2006; Zhou et al., 2013), and the processing of compound characters was modulated by features of the radicals (Feldman and Siok, 1997, 1999; Taft and Zhu, 1997; Taft et al., 1999; Ding et al., 2004; Lee et al., 2006; Wu et al., 2012). However, for the skilled Chinese readers, recognition of a compound character might depend more on the lexical representation. Most of the tasks for investigating the processing of Chinese characters, including the lexical decision task, always require a rapid decision to the linguistic stimuli, and as a result, the most critical sub-lexical information is supposed to be activated strongly and the less important sub-lexical information is to be suppressed in order to avoid a jam in cognitive processing. Based on this explanation, the absence of regularity effect in our study implied that pronunciation of the phonetic radical is a less important part of sub-lexical information. However, this inference seems to conflict with Wang (2006) which found a stronger effect of phonetic radical than that of semantic radical using the AM detection task. In our opinion, the stronger effect of phonetic radical found in Wang (2006) could be attributed to the orthographic superiority of phonetic radical. As the author argued, the phonetic radical was usually higher in size and lower in combinability than the semantic radical. In other words, the phonetic radicals were higher in complexity and variability (Wang et al., 2016). Moreover, the processing of characters in AM detection task is not naturalistic as either the semantic or the phonetic radial was displaced, and participants may focus more on the orthographic features of characters, so that the effect of phonetic radical may appear more prominent. Likewise, the stimuli employed in the present study also exhibited the systematic differences in these visual and orthographic features between the two types of radicals. Specifically, the phonetic radical had more strokes [*F*(1,274) = 193.56, *p* < 0.001, $\eta_{p}^{2}$ = 0.913], and was lower in combinability [*F*(1,274) = 242.71, *p* < 0.001, $\eta_{p}^{2}$ = 0.470] than the semantic radical. Although the phonetic radical has the superiority in orthography, the present study manipulating the functional validity of the radicals did not find any effect of phonetic radical. This suggested that, although the orthographic features of the phonetic radical might have a stronger influence than those of the semantic radical, the semantic radical might play a more important role at the functional level.

Interestingly, studies on alphabetic languages that employed a full factorial design also yielded different outcomes from those manipulating the variables of interest in separate experiments. For example, the latter type of studies which manipulated the frequencies of the two morphemes in alphabetic compound words had demonstrated that the first and the second morphemes could each play a role in the recognition of compound words (Duñabeitia et al., 2007). However, Duñabeitia et al. (2007), who factorially manipulated the frequencies of the two morphemes of both Basque and Spanish words in a lexical decision task, only found an effect of the second morpheme. Consequently, we assumed that manipulating the functional validity of semantic and phonetic radicals in a factorial design could allow us to compare the functional contributions of the two types of radicals. Anyway, the present study makes a theoretical contribution for us to further understand the processing of Chinese characters. The recognition of Chinese characters, a logographic script, was likely to depend more on the semantic route in a strategy-neutral task. Furthermore, some previous studies have revealed that Chinese children had mastered the semantic category concept of semantic radicals by Grade Three (Chan and Nunes, 1998; Ho et al., 2003), and the acquisition began as early as Grade One (Shu and Anderson, 1997).

Overall, the present results suggested that semantic radicals played a more important role in the recognition of phonograms than phonetic radicals. The composition of Chinese writing system, with radicals serving as an essential functional unit, is starkly different from the case of the alphabetical writing systems in which the words are formed of linearly arrayed letters. Alphabetical languages are characterized as having a grapheme-to-phoneme correspondence (GPC) so that each symbol is systematically mapped to a sound (Perfetti et al., 2007). It is more reliable to recognize an alphabetic word via a phonetically route instead of the semantic access path (Williams and Bever, 2010; Williams, 2012). As for a different case, the present results imply that as Chinese characters are a type of logographic script, the semantic route may be in more cases a default way when we attempt to recognize them. However, it is noteworthy that in the present study we only utilized SP phonograms, which were the most common used Chinese characters. So, it is quite necessary for additional studies to investigate the processing patterns with regard to phonograms in different structures, like the PS phonograms (which have the opposite configuration to SP phonograms, with the phonetic radical on the left and the semantic radical on the right) and even the top–down structured phonograms. Although we have no empirical evidence for the processing patterns of phonograms in other structures, we predict that the present conclusions can also be generalized to these phonograms if the functional validity of their semantic and phonetic radical are factorially manipulated. Moreover, although the lexical decision task benefited our study in that it is a strategy-neutral reading task allowing us to compare the processing of the semantic and phonetic radicals on a fair basis, it may not require substantial phonological activation of the host character as Yum et al. (2014) argued. This might explain why the present study failed to find any regularity effect. So further experiments can use other tasks, such as the semantic judgment task and the homophone judgment task, to test the generalizability of our results.

## Ethics Statement

This study was carried out in accordance with the recommendations of ‘Ethical Issues and Body Protection Guidelines in Psychology, the Ethics Committee of School of Psychology at Northeast Normal University’ with written informed consent from all subjects. All subjects gave written informed consent in accordance with the Declaration of Helsinki. The protocol was approved by the ‘The Ethics Committee of School of Psychology at Northeast Normal University.’

## Author Contributions

XW and YW contributed to the conception and design of the work. XW collected and analyzed the data. All authors contributed to discussion and writing of the manuscript.

## Conflict of Interest Statement

The authors declare that the research was conducted in the absence of any commercial or financial relationships that could be construed as a potential conflict of interest.
